# Effects of environmental translocation and host characteristics on skin microbiomes of sun-basking fish

**DOI:** 10.1098/rspb.2023.1608

**Published:** 2023-12-20

**Authors:** Hanna Berggren, Oscar Nordahl, Yeşerin Yıldırım, Per Larsson, Petter Tibblin, Anders Forsman

**Affiliations:** Ecology and Evolution in Microbial model Systems, EEMiS Department of Biology and Environmental Science, Linnaeus University, 391 82 Kalmar, Sweden

**Keywords:** freshwater, biodiversity, microbiota, skin microbiome, teleost, 16S amplicons

## Abstract

Variation in the composition of skin-associated microbiomes has been attributed to host species, geographical location and habitat, but the role of intraspecific phenotypic variation among host individuals remains elusive. We explored if and how host environment and different phenotypic traits were associated with microbiome composition. We conducted repeated sampling of dorsal and ventral skin microbiomes of carp individuals (*Cyprinus carpio*) before and after translocation from laboratory conditions to a semi-natural environment. Both alpha and beta diversity of skin-associated microbiomes increased substantially within and among individuals following translocation, particularly on dorsal body sites. The variation in microbiome composition among hosts was significantly associated with body site, sun-basking, habitat switch and growth, but not temperature gain while basking, sex, personality nor colour morph. We suggest that the overall increase in the alpha and beta diversity estimates among hosts were induced by individuals expressing greater variation in behaviours and thus exposure to potential colonizers in the pond environment compared with the laboratory. Our results exemplify how biological diversity at one level of organization (phenotypic variation among and within fish host individuals) together with the external environment impacts biological diversity at a higher hierarchical level of organization (richness and composition of fish-associated microbial communities).

## 1. Introduction

The ecological role of host-associated microbiomes is undisputable [1–5]. However, the factors that shape the composition and dynamics of microbiomes associated with aquatic organisms are still poorly understood, especially for skin microbiomes (electronic supplementary material, figure S1) [6]. For instance, within host species, there is often a large part of unexplained variation in microbiome composition among individuals [7–13]. That the composition of microbiomes varies among host individuals might reflect that they are shaped by highly dynamic interactions related to life stage [8,14,15] or diet [16], and perhaps also influenced by host genetics that may affect immune defence and mutualistic relationships [7,17,18]. However, little is known about if and how intraspecific variation in host phenotypic dimensions such as sex, morphology, behaviour and performance contribute to variation in microbiome composition, despite that these traits have bearing on both intrinsic and extrinsic factors that may impact the microbiome [19–24].

Studies of skin microbiomes on fish hosts are particularly interesting in this context. Because individuals vary in genetic composition, phenotypic attributes and behaviours, fish may be thought of as habitat islands with different properties that are colonized by microbial communities [22,25,26]. As fish move in search of food, mates, refuge and favourable temperatures [21,27,28], their associated skin microbiomes are constantly exposed to novel and changing external environmental conditions (e.g. different water depths, oxygen and light levels). Fish skin microbiomes thus offer an opportunity to investigate how biological diversity at one hierarchical level (among and within host individuals) impacts biological diversity at a higher level of organization (richness, composition and dynamics of microbial communities).

Extrinsic factors known to affect microbiome composition are water temperature, salinity, pH and UV radiation [15,29–31]. Aquatic host individuals experience differences in these factors as a result of habitat choice [19,20,22], sun-basking behaviour, personality and explorative behaviours [21,27]. Intrinsic factors, such as hormone secretion and physical properties of tissues, may also contribute to variation in microbiome composition among host organisms [32,33]. Both sex and colour morph are potentially relevant in this context, as typically being genetically determined, distinct and irreversible characteristics [34], that are associated with hormone levels [35], physiological differences and immune defence [23,24,33,36,37]. For instance, sex has been shown to mediate effects of dietary shifts on gut microbiomes in fish [38,39]. The association between intrinsic factors and among individual variation in microbiome composition is emphasized by a recent finding that growth was related to the gut microbiome in fish [40]. Despite their potential significance, associations of sex, colour, behaviour or growth with skin microbiome composition have rarely been investigated in aquatic organisms [41].

There are indications that fish skin microbiomes are highly environment dependent and hence likely more dynamic compared with gut microbiomes [13,42,43]. Little is known about the stability of microbiomes in a specific patch of an individual, but a previous paper reports that there was a high similarity of microbiome samples taken from corresponding spots on the left and right side of the same fish individuals, suggesting that fish skin microbiomes can be reliably assessed and characterized using a single sample from a specific body site [13]. There is also evidence from an experimental manipulation study of roach (*Rutilus rutilus*), based on repeated longitudinal sampling, that although fish skin microbial community composition can shift rapidly, even within a week, differences among fish individuals persist over time [42]. Regarding variation within individual hosts, different body parts may constitute micro-habitats with contrasting conditions [9,13,44]. For example, dorsal versus ventral parts of fish hosts may be differently exposed to sunlight (UV radiation), bottom sediment and abrasion, due to behaviours such as sun-basking, swimming activity and feeding strategy [20,45,46]. This can impact the microbiome community assembly processes [31,47,48] and contribute to differences in microbiome composition among hosts [9,11,49], yet it has rarely been investigated if and how body site of fish influence their skin microbiomes.

**Figure 1.**
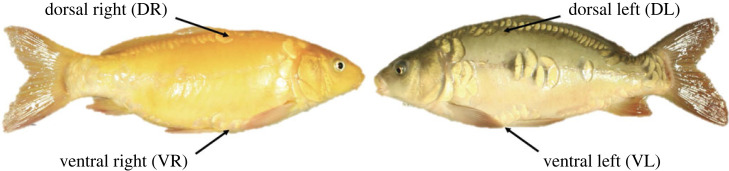
Colour morphs and body sites sampled. Behaviour and temperature data used in the study came from biologgers recovered from carp individuals that belonged to an orange (left) or a brown (right) colour morph. Before translocation to the pond, fish were sampled on dorsal and ventral body sites on the right side of the fish. At recapture, same body sites at the left side of the body were sampled. Photo by Oscar Nordahl.

To evaluate the roles of the external environment and host phenotypic characteristics on richness, diversity and community composition of the skin microbiome, we translocated carp (*Cyprinus carpio*) individuals from homogeneous captive conditions to a semi-natural heterogeneous environment and monitored their behaviour through laboratory trials and biologging (data storage tags). We hypothesized that after having spent time in the pond, microbiome composition should differ among host individuals, both according to body site and phenotypic dimensions. Specifically, we explored: (i) if and how richness, Shannon diversity and community composition of microbiomes changed following translocation of hosts from the laboratory to a semi-natural environment, (ii) if microbiomes collected from contrasting body sites (dorsal versus ventral) differed in community composition, (iii) if microbiome community compositions varied among individual hosts and (iv) whether and how the variation in microbiomes among host individuals was associated with host phenotypes (i.e. sex, colour morph) (figure 1), sun-basking, habitat switch (vertical and thermal), personality, body temperature and growth.

## 2. Material and methods

### (a) Study species, housing conditions and translocation procedure

In this study, we used common carp (*C. carpio*), a globally widespread cyprinid species of high cultural and socioeconomical value, due to its suitability of thriving in ponds, colour dimorphism and diverse behaviour relating to feeding strategies [50]. Carp is a rather long-lived species and frequently becomes older than 15 years [51]. The 48 fish in the study were 2 years old (mean length 28.7 cm, range 24–32 cm) and acquired from Aneboda Fiskodling, Sweden. Their colour morph was either brown (*n* = 25) or orange (*n* = 23; figure 1). Initially, these fish were housed in five freshwater tanks (600 l), randomly distributed based on colour morph and sex, at the Linnaeus University in Kalmar, Sweden, for approximately three weeks (14 May–8 June 2016) prior to translocation to the freshwater pond. Freshwater tanks were aerated and received regular tap water exchange. During the laboratory phases, carp were scored according to the boldness–shyness continuum with a standardized emergence test [52] to determine if they were consistent in behaviour over the study period. We performed a total of three emergence tests, two prior to and one post the period in the pond. All individuals were also equipped with biologgers (Lotek Lat 1410) that monitored depth, internal and external temperature every fifth minute (details in Nordahl *et al.* [27] regarding scoring of boldness and the surgical procedure for biologger implant). Carp were fed ad libitum with pellets during the laboratory phases, except for just prior to, and after, surgery. After three weeks in the laboratory, carp were translocated to a semi-natural (man-made) freshwater pond located 70 km north of the laboratory (lat. 57.173382, long. 16.032958, size 70 × 20 m, depth max 1.7 m) on 8 June 2016. Carp remained in the pond for two months and were recaptured via seine-haul fishing during 9–12 August 2016. The fish were transported back to the laboratory for repeated boldness assay, sex determination and removal of biologgers. Due to mortality and loss of biologgers, 27 individuals were used in this study (*n*-values for sex: brown/orange at recapture were as follows: 13 female: 7/6; 14 males: 10/4). Microbiome samples were taken in the laboratory just prior to the translocation and immediately in the field following recapture for each individual and before return to the laboratory.

### (b) Phenotypic data

To evaluate if microbiome composition was associated with host phenotypic variation, we extracted biologger data of water depth (cm) and temperature (°C) and body temperature (°C) from the last week before recapture (2–9 August 2016) and used these as estimates for behaviour and performance. This period was used because previous studies recently demonstrated that microbial community turnover in individual fish skin is rapid (within weeks rather than months) [10,42]. Information about host vertical migration and range in body temperature variation was used as a proxy for habitat switch (both vertically and thermally). We extracted the maximum and minimum in water depth and body temperature of each individual host during each hour of the last week in the pond and calculated a delta value (max–min) in cm and °C, for the two variables, respectively, that was summed up for the whole period. Sun-basking was measured as accumulated time of dormancy in the surface layer (measured in minutes) during periods of time categorized as basking (the fish had to be dormant in the surface layer for greater than 20 min during sunny conditions, see Nordahl *et al.* [27] for a detailed description about this definition). We used two different proxies for host physiological performance: growth (measured as weight gain during the whole period in the pond, mean = 221.4 g; range = 80–500 g), and the mean of the temperature excess acquired during periods of sun-basking for each individual compared with the water, *sensu* Nordahl *et al.* [27]. For the personality score, we used the mean emergence time from the two tests performed prior to, and the one test performed post-pond exposure.

### (c) Sample collection

We collected microbiome samples from dorsal and ventral body sites for each individual (*n* = 27) and environment (laboratory versus pond) representing 108 samples in total. In order to avoid the microbiome from being affected by previous sampling, the right and left side of the fish was sampled prior to, and post-translocation, respectively (figure 1). Before sampling of the microbiome, each fish was rinsed with MilliQ water to minimize the possibility of including loosely attached microbes belonging to the water column. The fish were then sampled on two different body sites, dorsal and ventral areas (2 × 2 cm) with a sterile cotton swab (Nordic Biolabs, CP167KS01) (figure 1). The swabs were put in Eppendorf tubes (1.5 ml) with 750 µl TE-buffer (Tris-EDTA, 10:1) and stored on ice until sampling was completed and then stored in a −80°C freezer. To avoid cross-contamination of microbiome samples, several precautions were taken between each sampling event: gloves were changed, touching the dorsal and ventral areas of fish avoided and the equipment used for sampling was disinfected with 70% ethanol between each sampling. Water samples (4 × 1 l) were taken from the pond at the day of release (8 June 2016) and at recapture (9 August 2016) for comparisons with fish skin microbiome samples. Information about DNA extraction and sequence processing can be found in electronic supplementary material, S2.

### (d) Data handling and statistical analyses

#### (i) Estimation of alpha and beta diversity

All statistical analyses were performed in Rstudio v.1.3.1093 [53] with R v.3.6.0 [54]. We included three alpha diversity estimates. Observed number of ASVs (100% identical ‘amplicon sequence variants') was used to illustrate the partitioning of ASVs according to sample type and environment. Statistical analysis of richness was based on estimates generated with default settings in the *breakaway* function (package breakaway v.4.6.11) [55]. To incorporate an alpha diversity measurement that take abundance and evenness into account we used the Shannon–Weaver diversity index [56] estimated from data subsampled to the smallest sample size (3775 reads per sample) using the *diversity* function in the vegan package (v.2.5-6) [57].

Apart from the subsampling prior to the Shannon index, raw data were used throughout the analyses. However, to explore the contribution of rare ASVs, we conducted a filtering step for comparison with the results based on raw data. To this end, we followed the method described in Bokulich *et al.* [58]: ASVs with a total count less than 10 within each sample and total abundance less than 0.01% across all samples were considered ‘rare’. This step decreased the total number of ASVs found in fish skin microbiomes from 16 881 to 1883 representing 11% of the total number of ASVs. The results based on data subsets are reported in electronic supplementary material, tables S2 and S3.

For beta diversity, the data were transformed by centred log ratio (clr) [59,60] allowing it to be used as input for linear regressions. Distances to group centroid for samples from each of the two environments were estimated from Euclidean distance matrix on clr-values, using the function *betadisper* (type = centroid) in the vegan package [57].

#### (ii) Investigating the effects of translocation, individual and body site on microbiome alpha and beta diversity with linear mixed models

We evaluated whether alpha (richness and Shannon) and beta diversity (measured as distance to Euclidean group centroid for each environment) varied among individual hosts, varied according to body site of the host (dorsal or ventral), changed over time, and whether shifts over time were different for dorsal and ventral body sites. To this end, we performed three linear mixed models with different response variables: richness, Shannon and distance to centroid (table 1). Richness and distance to centroid were both log-transformed to meet the assumptions for linear models. Body site (dorsal or ventral) and environment (laboratory or pond) were treated as fixed explanatory variables with an interaction between them, and individual was included as a random explanatory variable to account for repeated samples from the same individuals, and also allowing random intercept for different individuals [61]. Models were fitted by restricted maximum likelihood (REML) with the *lmer* function implemented in lme4 package (v.1.1-21) [62]. *p*-values were obtained with the *Anova* function in the car package (v.3.0-3) [63] using Wald *F* tests, Kenward–Roger degrees of freedom and type 3 sums of squares. Random effect of individual was evaluated with the *ranova* function in the package lmerTest (v.3.1-2) [64]. Results from these three models can be viewed in table 1.

**Table 1.** Effects of environmental translocation from laboratory to semi-natural habitat on alpha and beta diversity measurements. Here, results from linear mixed models are reported. Fixed and random effects were the same in all tests, only the response variable differed. Significant *p*-values are indicated in italic text.

response variable	independent variable	d.f.	test-statistics, fixed effects (*F*)	test-statistics, random effects (*χ*^2^)	*p*-value
richness	body site × environment	1, 78	2.43		0.12
	body site		9.44		*0*.*003*
	environment		30.2		*<0*.*001*
	individual		–	0.34	0.56
Shannon	body site × environment	1, 78	1.50		0.22
	body site		7.60		*0*.*007*
	environment		43.8		*<0*.*001*
	individual		–	2.15	0.76
distance to centroid	body site × environment	1, 78	2.51		0.12
	body site		11.6		*<0*.*01*
	environment		43.9		*<0.001*
	individual		–	0.005	0.94

To evaluate if there was a difference between body sites within each environment, we partitioned the data per environment (laboratory versus pond) and used paired *t*-test to account for samples taken from the same individual. We tested for homogeneity of variances between groups of multivariate data with the function *betadisper* in the vegan package [57].

To evaluate if community composition of microbiomes (*n* = 108) varied among individual hosts, according to body site (dorsal or ventral), changed over time and if temporal changes were individual specific, we performed a constrained redundancy analyses (henceforth RDA) [65] with the *rda* function in the vegan package. To account for repeated samples from the same individuals, we included individual as a conditional factor (which is similar but not equivalent to random effects in mixed models); conditioned factors are partialled out before analysing the effects of constraints [65]. Body site, environment, interaction between body site and environment and interaction between individual and environment were included as constraining variables. To test for differences in community composition between fish skin microbiomes and the bacterioplankton community in the water we performed an RDA on the full dataset (*n* = 116) with sample type as a constraining variable. Model fit for RDA analyses was evaluated with function *anova.cca* (settings: by = ’margin’ and default 999 permutations) in the vegan package. It is noteworthy that *anova.cca* will ignore main effects included in interaction terms [57,65].

#### (iii) Examining associations between host phenotypic variation and microbiome composition

We evaluated whether microbiome composition was associated with body site, sex, colour morph, behaviours (i.e. bold-shy personality, sun-basking and activity—as estimated by the switch between thermal and vertical habitats), growth and mean temperature excess obtained during basking. To this end, we used the microbiome samples collected after translocation to the pond, representing two samples per individual (one sample from each body site, figure 1) and performed an RDA. The explanatory data used in this analysis were based on the last week before recapture. During this last week, two individuals (females of each colour morph) did not exhibit any sun-basking behaviour and were accordingly excluded from the analysis and thus *n* = 50. To evaluate how much of the variation in microbiome composition that was explained by host individual alone compared with the phenotypic differences among the host individuals, we performed two analyses. The first model included body site and the eight phenotypic variables listed above as constraining factors. The second model included individual identity and body site as conditioned and constraining factors, respectively. Given the large number of explanatory variables included in the first model, we evaluated multicollinearity among the variables. To this end, we estimated the variance inflation factor (VIF) for each variable with the function *vif.cca* in the vegan package. The test suggested relatively low collinearity among variables (range = 1.00–2.97; table 2) given that VIF = 1 means that variables are independent and VIF > 10 is considered severe collinearity [65,66].

**Table 2.** Evaluating associations between microbiome composition and host phenotypic variation. Analyses on full dataset. Comparisons of results from the full model with all host phenotypic variables, and a simple model that included only individual identity as a conditioned factor together with body site as a constrained variable. In the full model, we evaluated whether microbiome composition was associated with any of the different host characteristics such as colour, sex, behaviour, performance and body site. The reduced model evaluated the effect of body site. Significant *p*-values are indicated in italics followed by an asterisk. n.a., not applicable.

variables included	variance partitioning	d.f.	test-statistics (*F*)	*p*-value	VIF
body site	constraining: 20%	1, 40	1.29	0.003*	1.00
basking			1.42	0.030*	2.97
vertical switch			1.28	0.033*	2.30
growth			1.36	0.032*	1.88
thermal switch			1.27	0.048*	2.40
temp. excess			1.19	0.152	1.88
personality			0.94	0.595	2.33
sex			1.10	0.262	1.98
colour			0.93	0.934	2.52
individual	conditioned: 50%	1, 24	n.a.	n.a.	–
body site	constrained: 2.6%		1.33	0.004*	–

#### (iv) Taxonomic analysis

To identify biomarkers (i.e. ASVs or taxonomic clades) associated with the skin microbiome pre- and post-translocation, or dorsal and ventral body sites post-translocation, we used the linear discriminant analysis (LDA) effect size (LEfSe) method described in Segata *et al.* [67]. LEfSe identifies taxonomic clades that are differentially abundant in contrasting biological conditions (here, laboratory versus pond environment) with non-parametric factorial Kruskal–Wallis rank sum test between environments and pairwise comparisons of each sample with Wilcoxon tests (alpha value for both tests was 0.05). Effects sizes were estimated with LDA such that each differentially abundant biomarker got an LDA score (log_10_ of abundance, threshold used = 4).

#### (v) Plots and graphics

All graphical output was produced in R [54]. Rarefaction curves, rank-abundance curves and heatmaps were generated with ampviz2 package (v.2.5.2) [68]. Venn diagrams were drawn with VennDiagram (v.1.6.20) [69]. Boxplots and PCA-plots/biplots were generated with ggplot2 package (v.3.3.3) [70].

## 3. Results

**Figure 2.**
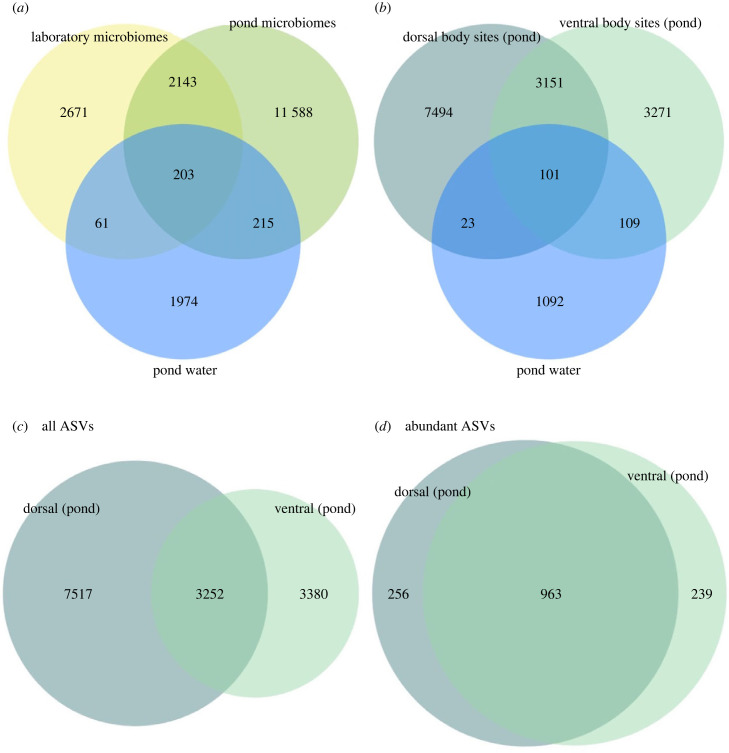
Venn diagram displaying the number of shared ASVs between bacterioplankton communities present in pond water and fish skin microbiomes. (*a*) Distribution of the total number of ASVs (*N* = 18 855) found among all skin microbiome (*n* = 108) and water samples (*n* = 8). (*b*) Post-translocation comparisons of the number of ASVs (*N* = 15 241) associated with dorsal (*n* = 27), ventral (*n* = 27) and water samples (*n* = 4), from the sampling after recapture in the pond. (*c*) Comparisons of all ASVs shared between dorsal and ventral body sites based on raw data comprising all ASVs detected among microbiome samples in the pond environment (*n* = 14 149) and, (*d*) filtered data, after excluding ASVs with a total count less than 10 within each sample and further with abundance less than 0.01%, leaving only the most abundant ASVs (*n* = 1458).

Overall, we identified 16 881 ASVs associated with fish skin microbiomes that were not detected in the water samples. A large proportion (approx. 89%) of these ASVs were less abundant than 0.01% across all samples and no ASV was detected in all samples. Translocation to the pond resulted in a major gain in number of observed ASVs among fish microbiome samples, from 4814 ASVs pre-translocation to 13 731 ASVs post-translocation (figure 2*a*,*b*). In the pond, the total proportion of sequences classified as rare ASVs increased to 40% from 14% in the laboratory, and they were to a high extent associated with dorsal body sites (figure 2*c*,*d*). The increased number of ASVs in the pond environment was not a blueprint of the bacterioplankton community present in the pond water: only 418 ASVs (2.6% of the ASVs sampled from water and skin microbiomes in the pond) were detected in both microbiome and bacterioplankton communities (figure 2*a*). The total number of observed ASVs was nearly seven times higher in skin microbiomes compared with bacterioplankton communities (16 881 versus 2453 ASVs associated with these two sample types, respectively, figure 2*a*). The community composition of the fish skin microbiome was clearly distinct from that in water samples (RDA: *F*_1,114_ = 4.92, *p* < 0.001) (see electronic supplementary material, figure S3).

### (a) Associations of alpha diversity with environment and body site

**Figure 3.**
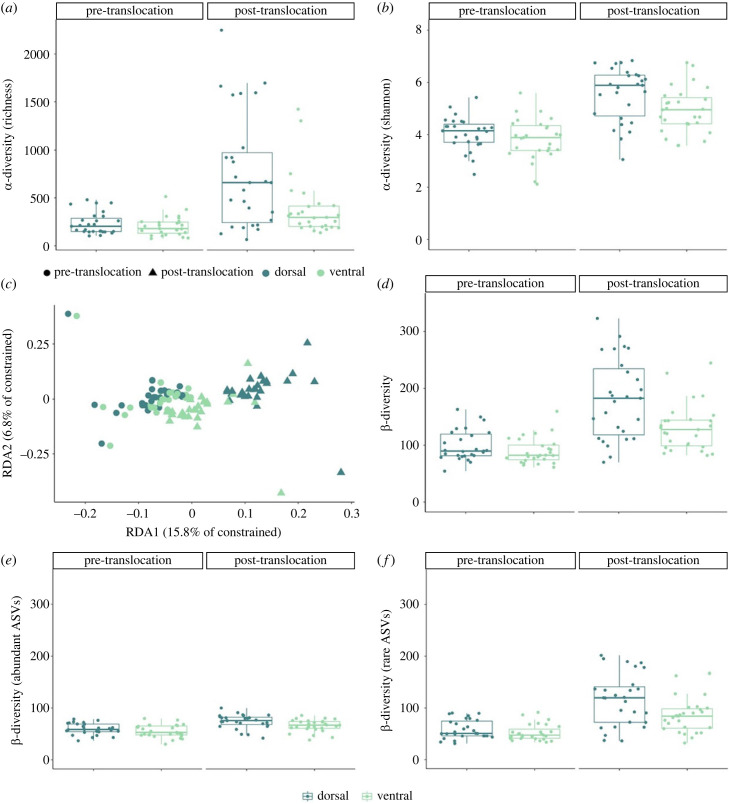
Alpha and beta diversity gain in dorsal and ventral fish skin microbiomes following translocation from laboratory to a semi-natural environment. Dorsal (teal) microbiomes diverged more than ventral (green) microbiomes following translocation. Alpha diversity, (*a*) estimated species richness and (*b*) Shannon diversity index. Community composition of skin-associated microbiomes, (*c*). Plot based on constrained RDA analysis evaluating interaction effects between environment (laboratory (circles) versus pond (triangles)) and fish individual, and between environment and body site. Beta diversity, (*d*) distance to Euclidean group centroid based on all ASVs (*n* = 16 881) detected among microbiome samples. Beta diversity (distance to Euclidean group centroid) based on (*e*) the most abundant ASVs (*n* = 1883) and (*f*) the rare ASVs (*n* = 14 999). All skin microbiome samples (*n* = 108) are included in each plot. Colours of the boxes indicate from which part of the body the sample was taken: dorsal (teal) or ventral (green). All data points are shown. Box-plot elements: centre line, median; box limits, upper and lower quartiles; whiskers, 1.5×interquartile range.

There was a significant effect of environment on skin microbial alpha diversity (linear mixed model, richness and Shannon: *p* < 0.01, table 1, figure 3*a*,*b*). Mean richness among microbiomes more than doubled during the period of free-ranging behaviour in the pond (mean ± s.e., laboratory: 219 ± 15; pond: 575 ± 69; figure 3*a*) and mean Shannon also increased among microbiomes during the two-month period in the pond (mean ± s.e., laboratory: 3.9 ± 0.09; pond: 5.2 ± 0.13; figure 3*b*). There was also a main effect of body site for both richness and Shannon diversity (linear mixed model, richness and Shannon: *p* < 0.001, table 1, figure 3*a*,*b*). Comparisons within each environment showed that the difference was only significant in the pond environment for both alpha diversity measurements (paired *t-*test for laboratory, richness: *t* = 1.35, d.f. = 26, *p* = 0.19; Shannon: *t* = 1.19, d.f. = 26, *p* = 0.24; pond, richness: *t* = 2.81, d.f. = 26, *p* = 0.009; Shannon: *t* = 2.91, d.f. = 26, *p* = 0.007; figure 3*a*,*b*).

### (b) Associations of beta diversity with environment and body site

For beta diversity (measured as the distance to group centroids for each environment using clr-based Euclidean distance), there was a significant main effect of both environment and body site, with beta diversity being higher post- than pre-translocation and higher in dorsal than in ventral body sizes, similar to the alpha diversity measurements (linear mixed model, *p* < 0.01, table 1, figure 3*d*). The main effect of body site was statistically significant only in the pond environment (paired *t*-test for laboratory: *t* = 1.52, d.f. = 26, *p* = 0.14; pond: *t* = 3.63, d.f. = 26, *p* = 0.001; figure 3*d*).

The community composition of skin microbiomes responded differently to translocation to a semi-natural environment depending on whether they inhabited dorsal or ventral body sites (RDA, effect of interaction between body site and environment: *F*_1,52_ = 1.28, *p* = 0.024; figure 3*c*). The interaction effect between environment and fish individual fell just short of the statistical significance level (RDA, effect of interaction between fish individual and environment: *F*_26,52_ = 1.05, *p* = 0.066). Analysing data from the laboratory and the pond separately revealed that the microbiome community composition of fish individuals and body sites did not differ significantly before but after being translocated to the pond (RDA for laboratory samples, effect of individual: *F*_26,26_ = 1.02, *p* = 0.18, body site: *F*_1,26_ = 0.95, *p* = 0.68; RDA for pond samples: effect of individual: *F*_26,26_ = 1.05, *p* = 0.001, body site: *F*_1,26_ = 1.33, *p* = 0.003). This means that the variation in microbiome composition increased when fish were exposed to the semi-natural environment (test for homogeneity of multivariate dispersions in each environment: *F*_1,106_ = 45.3, *p* < 0.001, figure 3*c*,*d*).

### (c) Taxonomic turnover in fish skin microbiomes following translocation

The 12 most abundant phyla among both water and skin microbiome samples represented 96% of the total number of sequences (see electronic supplementary material, figure S3). Proteobacteria, Actinobacteria and Bacteroidetes were the three most abundant phyla in both sample types and represented 94% of all sequences among water samples, but only 68% of all sequences among microbiome samples.

The high number of ASVs detected in skin microbiomes from the pond was positively associated with the number of phyla (see electronic supplementary material, S3 and figures S4, S5). The number of detected phyla in skin microbiomes increased from 44 to 59 following the translocation to the pond. There were seven phyla present in all microbiome samples: Acidobacteria, Actinobacteria, Bacteroidetes, Firmicutes, Planctomycetes, Proteobacteria and Verrucomicrobia. Phyla detected in the laboratory were also detected in the pond and the four most abundant taxonomic groups were the same after translocation: Proteobacteria (classes Gamma- and Alpha-proteobacteria), Bacteroidetes and Actinobacteria (electronic supplementary material, figure S6*a*,*b*).

There were 8 and 12 significantly enriched taxonomic groups in the laboratory and pond environment, respectively (identified by differential abundance analysis performed with LEfSe and depicted with black asterisks in electronic supplementary material, figure S6 [71]). A similar comparison between the body sites post-translocation indicated that no taxonomic groups were associated with dorsal body sites and only two with ventral body sites: Actinobacteria, family *Microbacteriaceae*, and Alphaproteobacteria, genus *Sphingomonas* (red asterisks in electronic supplementary material, figure S6; [71]). The ASVs classified as abundant covered 34 phyla and the rare ASVs represented the same phyla plus an additional 25 phyla.

### (d) Shifts in microbiome composition were associated with variation in host phenotypes

Body site, time spent sun-basking, growth, vertical and thermal habitat switch were all significantly associated with the variation in community composition among individuals in the pond environment (RDA: *p* < 0.05, table 2, electronic supplementary material, figure S7), whereas mean temperature excess, personality, colour and sex were not. When all constraining variables were included in the same model they explained 20% of the total variance. By contrast, results from the model that only included individual identity and body site explained 53% of the total variance (table 2).

Analyses and comparisons of microbiome composition based on subsets of the data, either the most abundant ASVs (accounting for 11% of the members in the community) or only the rare ASVs (accounting for 89% of the members in the community), generated different results. The rare ASVs were significantly associated with body site, sun-basking, vertical activity, growth and thermal habitat variation among individuals (electronic supplementary material, table S2). The abundant ASVs were not associated with any of the host characteristics, instead they were significantly associated with body site (electronic supplementary material, table S3).

## 4. Discussion

Assessing host-associated microbiomes *in situ* can help identify external environmental as well as individual-specific factors that influence microbiota transmission, acquisition and functions, in their natural ecosystems [72,73]. Here, we investigated if and how environmental translocation, body site and phenotypic variation among fish individuals affected their skin microbiome. Overall, the results supported our hypothesis that after having spent time in the pond, microbial composition should differ among host individuals, both according to body site and phenotypic dimensions. Specifically, we found that following translocation of the fish hosts from captive laboratory conditions to a semi-natural environment, alpha and beta diversity increased markedly within and among individuals, and particularly on dorsal body sites. The results also showed that the microbiome composition was distinct from the bacterioplankton community in the pond water and associated with five of the nine measured phenotypic host traits; body site, sun-basking time, habitat switch (vertical and thermal) and growth. These findings are discussed and described in greater detail below.

### (a) Number of ASVs and phylogenetic diversity increased following translocation

Translocation from laboratory conditions to a semi-natural environment induced a threefold ASV gain in the skin microbiome of fish individuals and involved an increase from 45 to 59 phyla. The ASVs gained following translocation did not mirror the taxonomic composition of the bacterioplankton in the pond water, indicating that fish hosts are important habitats for epibiotic microbial communities in natural habitats. The microbial variants found on fish skin but not in the pond water could either be due to that the strains were too rare for detection, or that they originated from the pond sediments or other biofilms, but we did not collect any such environmental samples in this study so the conclusions must be tentative. A strong link between fish microbiome and environmental microbial communities has been found in hatchery reared fish [74], but a recent study on wild caught fish, representing over 100 species, that included several environmental source samples concluded that the majority of microbiome members were of unknown origin and not present in the water column or bottom sediment [6].

The observed alpha and beta diversity gain following translocation to a natural environment is in agreement with previous studies [10,17,75] and it has been suggested that higher microbiome diversity in terms of both richness and community composition in natural conditions is a reflection of a more heterogeneous and changing environment, which may impact both the pool of potential colonizers and exposure to stressors [17,75–77]. This may partly explain the patterns observed in this study, but it was also evident that microbial richness and community composition varied to a greater extent among fish individuals post-translocation. The potential role of host age for microbiome diversity, as have been suggested for both human and fish gut microbiomes [78,79] also do not provide a satisfactory explanation for the increase in alpha and beta diversity post-translocation because variation increased among individuals that were of the same age. A possible explanation for this increased variation is that the phenotypic and behavioural variation among host individuals was exaggerated under the more natural and heterogeneous conditions in the pond [20]. If so, the potential for variation in host phenotypes (and the external conditions to which they are exposed) to impact the skin microbiome may have increased in the semi-natural environment.

### (b) Associations of microbiome composition with host behaviours and growth

We investigated a suite of phenotypic dimensions of carp to shed light on how individual variation in phenotypic traits of the hosts may be associated with community composition in skin microbiomes. Interestingly, behavioural differences among host individuals in habitat switch (vertical and thermal) and sun-basking behaviour, but not boldness, was associated with more variable skin microbiomes. To our knowledge, associations of these behavioural traits with skin microbiomes in fish—or in other aquatic organisms—have not previously been investigated, although a recent study found that, across species of fish, alpha diversity was negatively associated with swim endurance [6]. When fish switch in vertical and thermal habitat, they are exposed to changes in various environmental conditions (e.g. temperature, pH, oxygen, UV radiation and dissolved organic material) that can impact the skin microbiome [30,31,47,80,81]. The vertical habitat switch may reflect times when the dorsal part of the fish approaches the water surface and sunlight, while when moving downward, the ventral part of this benthically feeding fish comes in contact with the bottom sediment and is exposed to abrasion and a different bacterioplankton community. We suggest that higher activity rate will increase the probability for the fish to encounter potential colonizers which could then lead to diversification of microbiomes among individuals. That the variation in microbiome composition was associated with sun-basking behaviour when the fish are dormant in the surface waters during sunny conditions *sensu* Nordahl *et al.* [27] could have been caused by direct exposure to UV radiation which is known to have a significant effect on microbial communities [80]. This interpretation is strengthened by the comparisons of the composition of microbiomes associated with dorsal and ventral body sites in the pond and in the laboratory, as outlined below. Differences in skin microbiome composition were also associated with host growth rate which may reflect an adaptive value similar to what has recently been found regarding the gut microbiome of fish [82]. However, our conclusions must be tentative; to establish the directionality and causality of the association with growth rate would require manipulation experiments.

### (c) Microbiomes on dorsal body sites were more affected by translocation

Microbiomes were more diverse on dorsal than ventral body sites after two months of free-ranging in the pond. For instance, the second LEfSe analysis that compared abundance of taxonomic groups between the body sites, only found two enriched groups and these were associated with ventral body sites, supporting the notion that variation in community compositions was greater for dorsal body sites. Specifically, we found that rare (and potentially transient) ASVs inhabited dorsal body sites to a larger extent. Exposure to UV radiation can cause disturbances that affect the colonization–extinction dynamics [83] and alter community composition [84]. Ecological theory posits that variation in microbiomes among hosts should increase with disturbance frequency in patchy environments [85–88]. Depending on the frequency and magnitude of disturbances, the effect on the community can be substantial [89], and compounded perturbations can result in unpredictable outcomes [90]. More frequent disturbances could also allow for higher immigration rates from the surrounding bacterioplankton communities [91]. Indeed, that microbiomes associated with dorsal body sites varied more than the ventral microbiomes indicates that different processes were involved. As such, the lack of association with host body temperature achieved during sun-basking further supports that it was the exposure to sunlight/UV radiation *per se* (not body temperature) that contributed to the differences between dorsal and ventral microbiomes.

### (d) No association of microbiome composition with sex or colour morph

Our results did not point to any effects attributable to either sex or colour morph. Sex-specific effects have been reported in both skin and gut microbiomes of humans and mice [24,33,92], and are suggested to covary with sex-dependent hormones affecting bacterial growth rate, but also physiological differences in the gut region according to sex. A few previous studies report on sex-dependent responses of fish gut microbiome to changes in the diet [38,39], whereas another study found no effect of sex on the assembly of fish gut microbiomes [93]. Also, colouration has been shown to be associated with microbiomes in scallops [94]. Further studies are clearly needed to draw firm conclusions about the roles of sex and colouration on skin microbiome composition. Admittedly, our findings suggest that much of the variation (26%) in microbiomes among host individuals could not be accounted for by the phenotypic variables that we measured, compared with the proportion variation explained when host individual was included in the analysis (53%). This could indicate either that other host traits, such as individual variation in the immune system [7,95,96], are important drivers of the microbiome, or that the colonization and extinction of skin microbial communities are largely influenced by random events.

### (e) Associations were different for abundant and rare microbiome members

The comparisons of results from the analyses of rare and abundant ASVs indicated that the different body sites were affected by different assembly processes. The results based on the analysis of rare ASVs (89% of the microbiome community) indicated a stronger association with behavioural and physiological host phenotypic dimensions than the whole dataset, whereas the most abundant ASVs (11% of the microbiome community) were associated with body site but not with phenotypic dimensions. The different results and conclusions emphasize the potential risk of rarefying data before analyses are conducted, because ecologically important patterns might be overlooked [73,97]. For instance, Boutin *et al.* [98] isolated probiotic strains from fish skin microbiomes and these strains were not among the most abundant microbiome members (less than 0.1%). Thus, rare microbiome members may also have important functions and a potential value as probiotic strains for aqua and agriculture [98,99].

## 5. Conclusion and future directions

A major conclusion that emerges from our results is that diversity at one level of biological organization (phenotypic variation among and flexibility within host individuals) seems to carry-over and support an increased biological diversity also at another, higher, hierarchical level of organization (richness and species composition of host-associated microbial communities). Host individuals in natural conditions vary more in their behaviour and are subsequently exposed to varying environmental conditions which results in larger variation in microbial alpha and beta diversity among hosts. This adheres to a growing body of evidence from studies of other systems that larger intraspecific trait variation of plants and macroalgae can support more species rich and diverse communities at higher levels of organization (e.g. [100,101]). One way to further evaluate this hypothesis would be to compare richness and diversity of external (or internal) microbiomes in groups of hosts that are genetically and phenotypically more or less variable. Another striking finding of this study was that many of the flexible behavioural and physiological host traits were associated with microbiome communities whereas the two structural (and irreversible) traits were not. Yet, most of the variability in skin microbiomes among hosts could not be accounted for by the suite of phenotypic properties that we measured, suggesting that the assembly, structure and dynamics of fish skin microbiomes are influenced by a large number of interacting factors. Future studies should investigate additional host traits and evaluate the role of stochastic processes for this striking diversity. The realization that fish skin microbiomes represent biodiversity hotspots that vary according to host characteristics and change over time emphasizes the need to aid the protection of such systems. Further studies are also motivated by the huge socioeconomic importance of fish worldwide [102,103], because the associated microbes may impact host well-being and ultimately food productivity [104–107]. In this context, investigating the different functional pathways (e.g. response of host immune system) by which the microbiome may influence host performance and fitness will remain an important task for future research.

## Data Availability

Data available from the Dryad Digital Repository: https://doi.org/10.5061/dryad.w9ghx3fw7 [71]. The raw sequences are available in the NCBI's Sequence Read Archive (SRA) under BioProject's PRJNA714685 and PRJNA673155. Supplementary material is available online [108].
